# Towards a Low-Cost Monitor-Based Augmented Reality Training Platform for At-Home Ultrasound Skill Development

**DOI:** 10.3390/jimaging8110305

**Published:** 2022-11-09

**Authors:** Marine Y. Shao, Tamara Vagg, Matthias Seibold, Mitchell Doughty

**Affiliations:** 1 Centre For Print Research, University of the West of England, Coldharbour Lane, Bristol BS16 1QY, UK; 2 Cork Centre for Cystic Fibrosis (3CF), Cork University Hospital & HRB Clinical Research Facility Cork, University College Cork & School of Computer Science and Information Technology, University College Cork, T12 XF62 Cork, Ireland; 3 Computer Aided Medical Procedures (CAMP), Technical University of Munich, 85748 Munich, Germany; 4 Research in Orthopedic Computer Science (ROCS), University Hospital Balgrist, University of Zurich, 8008 Zurich, Switzerland; 5 Department of Medical Biophysics, University of Toronto, Toronto, ON M5S 1A1, Canada

**Keywords:** augmented reality, cross-sectional anatomy, medical ultrasound, ultrasound education

## Abstract

Ultrasound education traditionally involves theoretical and practical training on patients or on simulators; however, difficulty accessing training equipment during the COVID-19 pandemic has highlighted the need for home-based training systems. Due to the prohibitive cost of ultrasound probes, few medical students have access to the equipment required for at home training. Our proof of concept study focused on the development and assessment of the technical feasibility and training performance of an at-home training solution to teach the basics of interpreting and generating ultrasound data. The training solution relies on monitor-based augmented reality for displaying virtual content and requires only a marker printed on paper and a computer with webcam. With input webcam video, we performed body pose estimation to track the student’s limbs and used surface tracking of printed fiducials to track the position of a simulated ultrasound probe. The novelty of our work is in its combination of printed markers with marker-free body pose tracking. In a small user study, four ultrasound lecturers evaluated the training quality with a questionnaire and indicated the potential of our system. The strength of our method is that it allows students to learn the manipulation of an ultrasound probe through the simulated probe combined with the tracking system and to learn how to read ultrasounds in B-mode and Doppler mode.

## 1. Introduction

Ultrasound is an important diagnostic modality and is capable of producing images of tissue, organs, or blood flow via high-frequency acoustic energy transmitted to the human body via the skin [1]. Traditionally, sonography is taught and certified at postgraduate level (2–4 years on average but it may take longer for certain specialities) [2].

During these postgraduate courses, ultrasound education is divided into two areas: (1) lectures focusing on the theory, physics, and image acquisition; and (2) practical exercises designed to develop psychomotor skills [3]. These practical sessions can include supervised simulator use, or clerkship experiences with patients [4]. In a recent review, Pessin et al., found that 75% of respondents used simulation as part of their ultrasound education curriculum [5]. Further, it was reported that simulation was a useful teaching tool for sonography (89%) and resulted in a highly positive student experience (81%) [5].

In recent years, the introduction of reduced form factor ultrasound devices has improved convenience and accessibility for educational and diagnostic purposes. Further, ultrasound education is increasingly being introduced at undergraduate level as part of medical anatomy training with a greater emphasis on students to continue practicing their ultrasound training at home. This need has led to creative low-cost home-made phantom devices using easily accessible materials such as tofu, gelatine, and spam [6,7,8,9]. However, continued home-based training requires the student to have access to an ultrasound device, which is not always possible. The limited- or no-access to digital devices required for education and training also lends itself to a larger discussion of digital exclusion, whereby those with unequal access to the technology are at a disadvantage [10].

The ongoing COVID-19 pandemic has necessitated significant structural changes to primary, secondary, and tertiary education, with most programs now being taught online in “virtual classrooms”. Particularly for ultrasound education, classroom-based lectures have transitioned to become virtual, and practical sessions have been postponed or annulled [11]. In some instances, innovative digital solutions were implemented to promote remote ultrasound education. This is particularly evident in Meuwly et al., study [12] which employed an online simulator for basic psychomotor skills education during the pandemic, resulting in substantial improvements in the psychomotor field of ultrasound diagnosis, while this solution still requires students to have access to a personal computer and internet, it removes the necessity for an ultrasound device and maintains a strong learning outcome [12].

### 1.1. Motivation

We propose the development of a self-led at-home training solution enabling students to learn the basics of ultrasound imaging and ultrasound probe manipulation through an augmented reality (AR) solution. To guide the learner, we map and register elements of the real environment to virtual training elements environment of the student in real-time and interact with it in a virtual setting displayed on a computer. The requirements for a student to use this system are: (1) a computer with webcam; and (2) a printed ArUco marker.

### 1.2. Related Work

In the context of ultrasound education, the effective use of AR is limited. In 2015, Palmer et al., developed a mobile AR system (SmartScan) to facilitate wider adoption of ultrasound education and to quicken the learning process [13]. The mobile AR system displayed a virtual heart within a patient’s body using AR and data provided by an ultrasound probe. To correctly align the virtual and real information, a fiducial marker was added to the patient and probe. In their manuscript, the authors did not present any initial evaluations of the system. Another similar manuscript published by Mahmood et al., in 2017 suggested that AR for ultrasound education benefits educators by allowing them to teach imaging techniques with a focus on enhancing learned spatial orientation [14]. In this study, the authors used a Microsoft HoloLens in conjunction with a Vimedix transesophageal echocardiogram (TEE)/transthoracic echocardiogram (TTE) simulator. The authors did not provide any details of a formal evaluation.

Meaningful AR centers on the generation of a virtual experience that is informed by relevant elements of the current real scenario of a user and enables interactions between the real and virtual world. A requirement of an AR system is the position and orientation (pose) tracking of elements in the environment of the user or of the user itself. In the context of medical education, tracked elements can include instruments or surgical phantoms [13]. In the computer vision community, there exist two primary strategies to track the 3D position of humans: (1) using a two stage approach based on a 2D body joints pose estimation followed by lifting in 3D space [15]; or (2) using a one stage approach using volumetric heatmaps [15].

## 2. Materials and Methods

**Figure 1:**
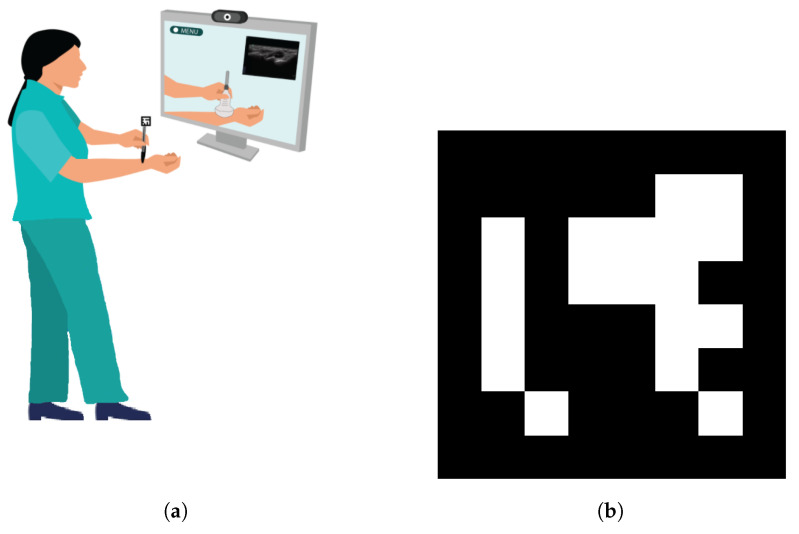
Ultrasound training system (**a**) using the ArUco marker (**b**).

The concept of the developed home-based training system is presented in Figure 1. The system provides context based instructional content on the basics of how to use an ultrasound probe and how to interpret ultrasound image data. The learner is not required to have access to an ultrasound probe and can mimic it with a pen or any other object with a similar geometry as an ultrasound probe. The simulated ultrasound probe is then tracked with an affixed square ArUco marker which is printed on a piece of paper. The arm of the learner is tracked with 3D RGB body tracking. As the learner is not using an ultrasound device, the ultrasound images displayed are pre-recorded and are related to the current learner anatomy using a proximity based approach with several defined landmarks across the arm. To accelerate further investigations in this space, we have made our source code publicly available on GitHub at the following link: https://github.com/doughtmw/AR-Ultrasound-Training (accessed on 18 October 2022).

### 2.1. Training Process Overview

During a training session, a learner is guided through the following process:

1. The learner is positioned in front of the computer,
2. The learner launches the Unity scene on the computer; the live video from the learner recorded by the webcam appears—the whole body must be visible for the process to work,
3. Once the green skeleton following the movement of the learner is loaded, the learner is instructed to follow a registration process to adjust the precision of tracking by placing an ArUco marker at four different joints: left shoulder, left elbow, let hand, and right hand. During this process, detailed instructions for the registration are displayed on the screen to the learner.
4. The learner then places one ArUco marker on the simulated probe. On the screen of the computer, the learner can see the simulated space where a tracked virtual arm and virtual ultrasound probe will appear. If registration has been completed successfully, the virtual arm will follow the arm of the learner thanks to the body tracking; additionally, the virtual ultrasound probe will follow the simulated probe thanks to the ArUco marker.
5. The learner is then instructed to select on one of the ultrasound buttons on the screen. An ultrasound display screen will appear. The learner can then choose to perform the simulated ultrasound scan in B-Mode or in Doppler mode.
6. The learner is instructed to begin the examination by running the simulated probe along their arm. During this process, the ultrasound images will appear on the ultrasound display screen—these images are pre-recorded on the arm of a consenting volunteer. The learner is freely available to move the pen/probe and investigate their local anatomy as represented by the virtual information on the computer monitor.

### 2.2. Images Recording

The training system relies on pre-recorded 2D ultrasound images; we recorded the data using the Healcerion Sonon 300L ultrasound probe on a consenting volunteer. All the data comes from the same healthy volunteer; to our knowledge, there is no specific pathology within the images.

**Figure 2:**
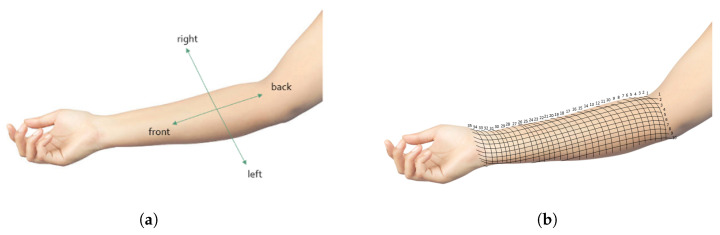
Direction of the angles in the image acquisition (**a**) and positions of acquisitions points during the image acquisition (**b**).

The images were recorded on a predefined set of points on the forearm of the volunteer. The points are located at the intersections of two grids. The first one divides the arm from the interior of the elbow to the wrist at 35 regularly distributed positions, as represented in Figure 2. The second one divides the arm from right to left at 10 regularly distributed positions. Due to the importance of the control of the angle between the probe and the arm in order to get the best view during an ultrasound examination, images were recorded by manually sweeping the probe from back to front (−β,β) at each of these points. The images were recorded by taking a short video while moving the probe. The sample ultrasound data set consists of 40 frames at each point.

The images were recorded in two different conditions to provide more training modes to the student. The first recording condition involved B-Mode in the musculoskeletal setting with a depth of 4 centimetres and a frequency of 10 Mhz. The second recording condition involved Doppler mode in the musculoskeletal setting with a depth of 4 centimetres and a frequency of 10 Mhz.

In Doppler mode, the image can change while staying at the same position with the same angle because of the dynamic blood flow; for this reason, we additionally recorded two short videos of 10s including a Doppler scan at two fixed positions. These videos are also added in the training modes to see how the Doppler images can vary over time.

### 2.3. Tracking

The tracking of the marker and arm of a learner is performed with Unity 2020 LTS, Visual Studio, Visual Studio Code, and Python 3.9 for Windows.

#### 2.3.1. Aruco Tracking

The simulated ultrasound probe held by the student is tracked using an ArUco marker [16]. The ArUco marker is printed on a piece of paper. The marker is detected by the webcam through the following process:

1. The first step was the segmentation of the image to extract the contours. The method used for the segmentation is the local adaptive thresholding approach; it results in a binary image.
2. The second step aimed to identify the contours of the markers. After the segmentation, the contours of the binary image were approximated using polygonal approximation. Because the ArUco markers have a rectangular shape, only the contour made of a 4 vertex polygon are selected.
3. The contents of each extracted contour were then analysed. The first step is the removal of the perspective projection in case the marker was not facing the camera. Then the interior of each contour was binarized and divided into a regular grid. At last, we assigned a value of 0 or 1 to each cell of the grid. This step allows the identification of a list of marker candidates.
4. Each marker candidate obtained in stage 3 are compared to the ArUco marker library. We tested each possible rotation to avoid missing a marker. The aim was to detect the ArUco markers recorded by the webcam.

Once the marker is detected, its position in the space is estimated using an iterative process aiming to minimise the reprojection error of the position of the corners [16]. During this step, the size of the printed marker is known by the algorithm.

Another commonly used tracking method is optical tracking; it is also based on cameras and markers; however, the detection of the markers is not based on the same technique [17]. With optical tracking, the markers are reflective and the detection technique is based on the reflection of the light on the markers. The system includes multiple cameras and the position of the marker is calculated from the reflected light. With our method, the markers are detected through image analysis and detection of patterns. The strength of the method is that it only requires markers printed on paper and a single camera.

#### 2.3.2. Rgb-Based Human Pose Tracking

**Figure 3:**
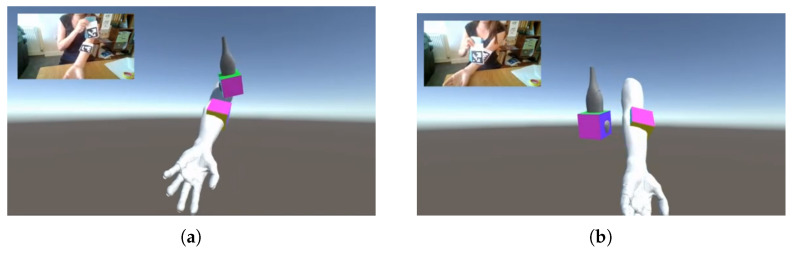
Issue of occlusion when using two ArUco markers for the tracking. (**a**) Tracking of the arm and the ultrasound probe using ArUco markers; no occlusion. (**b**) Tracking of the arm and the ultrasound probe using ArUco markers; occlusion of the arm marker by the probe marker and impact on the tracking of the arm in the virtual scene which no longer follows the arm of the student.

Simultaneous tracking of the arm and the simulated probe with ArUco markers may lead to an issue due to the occlusion of one of the markers, as visible in Figure 3b. To facilitate the use of the proposed system, an alternative solution was implemented to track the arm of the student using deep learning-based human pose regression. This approach has the advantage of requiring only a single marker for the tracking of the simulated ultrasound probe and increases the usability of the proposed training application. Furthermore, no additional hardware is required, as state-of-the-art methods exist that infer the 3D locations of the human body joints directly from a two-dimensional RGB input image.

Even though RGB-based human pose tracking is less accurate than approaches which incorporate depth information, the accuracy is sufficient for the given application. There are two different approaches for inferring the 3D locations of the human body keypoints from RGB images. The first one is to detect the 2D locations of the keypoints in the image space and subsequently lift these 2D coordinates to the 3D space [18]. The second approach is to directly regress the 3D human keypoint locations from an RGB input image. VNect [19] directly regresses the 3D human keypoints and refines the full global 3D skeletal pose using a kinematic skeleton fitting step in a temporally consistent manner.

Our implementation utilized the Unity Barracuda ONNX framework to run a pre-trained VNect model for inference directly in Unity [19]. Together with the marker tracking for the simulated ultrasound probe this formed the foundation for the proposed training application; however, our system required an additional calibration step to relate the tracking coordinate systems of the ultrasound marker and arm of a learner which is described in detail in the following paragraph.

### 2.4. Calibration of the Tracking

The system included two tracking methods: (1) RGB human pose; and (2) ArUco marker tracking. Given a reference point *X*, the coordinates of *X* in the basis of the ArUco tracking (BA) was XA and the coordinate in the body tracking basis (BBT) is XBT; XA and XBT were (3,1) vectors with the values of the x,y,z coordinates of *X* in each basis. In order to compare the position of the probe detected by the ArUco marker to the position of the arm detected with the RGB body tracking, the coordinates must be expressed in the same basis.

Because of the two different tracking methods based on ArUco tracking and RGB tracking, the system required a calibration procedure to run correctly. The calibration determines the (3,3) translation matrix RBTA from the basis BA to the basis BBT. Because the origins of both basis also differ, the calibration also requires a translation vector between the two basis TBTA. The position of *X* in the basis BA can the be calculated by:

(1) XA=RBTA·XBT+TBTA

Equation (1) expresses the coordinate of a point *X* in the basis BA from its coordinates in the basis BBT.

The calibration procedure guides the learner through registration of the coordinates of 4 points: $X_{1}$, $X_{2}$, $X_{3}$, and $X_{4}$ in both basis systems by superimposing the ArUco marker to the joints of the user. The targeted joints are the left shoulder, the left elbow, the left hand, and the right hand; these joints were selected as they provide consistent key points to track during the training session as the learner practices scanning of their left arm.

Once the coordinates of the 4 points are determined, the following procedure is followed to calculate the translation matrix:

(2) TBTA=X1A−RBTA·X1BT

Equation (2) express the vector TBTA in function of the coordinates and the matrix RBTA. If we define the vector V = (1, 1, 1). Then, by combining Equations (1) and (2):

(3) AX2AX3AX4=RBTAX2BTX3BTX4BT+X1A·V−RBTA·(X1BT·V)

Then:

(4) AX2AX3AX4=RBTAX2BT−X1BTX3BT−X1BTX4BT−X1BT+X1A·V

Because the points $X_{1}$, $X_{2}$, $X_{3}$, and $X_{4}$, are not in the the same plane, the column of the matrix X2BT−X1BTX3BT−X1BTX4BT−X1BT are independant and the matrix is invertible. As such, we can the express the translation matrix as follows:

(5) RBTA=AX2−AX1AX3−AX1AX4−AX1X2BT−X1BTX3BT−X1BTX4BT−X1BT−1

### 2.5. Ultrasound Display

The webcam records the live scene, computes the pose of the ArUco marker in frame and updates the position of the probe in the simulated space. The webcam also estimates the body of the learner using RGB tracking and updates the position of the arm in the simulated space accordingly.

When the ultrasound images are displayed on the screen, the choice of the ultrasound image to display depends on the distance between the the marker and the left elbow of the student. If *L* is the distance between the wrist and the elbow of the arm in the simulated place, *n* is the number of ultrasound images $\left[i_{1},i_{2},\ldots ,i_{n}\right]$, and *l* the distance between the two markers, then the number of the image being displayed *i* is described as:

(6) $$i=\frac{l}{L}\times n$$

Because *L* is user specific, it is calculated for each user during the registration step where the student places the marker at different body joints locations.

**Figure 4:**
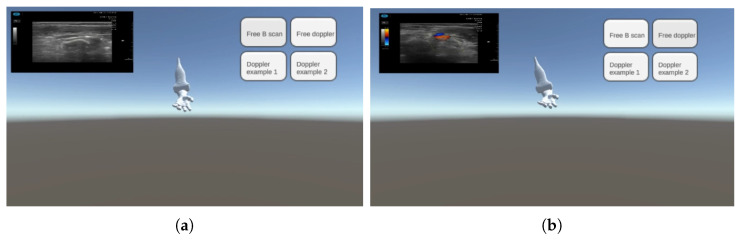
Screenshot of the Current System when training in B-Mode (**a**) and in Doppler mode (**b**).

Similarly, the angle between the simulated probe and the simulated arm is computed and based on the result, an ultrasound image specific to the angle is displayed. As visible in Figure 4, the displayed depends on the training mode chosen by the student.

### 2.6. Evaluation

A group of 4 experienced medical ultrasound lecturers were recruited to test the system. All participants were from the same center; they were recruited with written consent according to the local regulation. Prior to testing the system, the participants were briefed on the aim of the study and shown a live demonstration on how to use the system. The briefing included an introduction to ultrasound education and simulation and a presentation of the system. The participants were then asked to test the system and evaluate their experiences by answering a survey.

## 3. Results

Our technique updates the position for each new video frame that is received. This update consists of (1) GPU based network inference of the model used for marker-free body pose estimation; and (2) CPU based square marker tracking with the ArUco library and traditional computer vision techniques. It requires 15% CPU (3.5 GHz), 65% memory (1800 Mb), and 85% GPU (2.5 GB) on a consumer-grade computer with a Quadro P600 graphic card, ® Core™ i7-8850H CPU @ 2.60 GHz CPU, and 16.0 GB of RAM.

**Figure 5:**
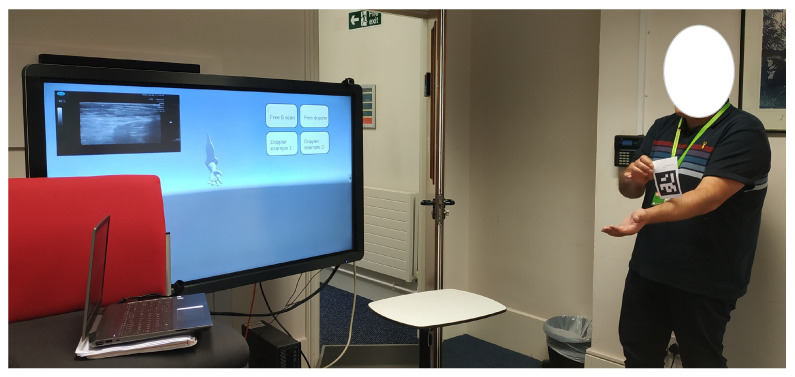
Participant testing the ultrasound training system.

As visible in Figure 5, 4 medical ultrasound lecturers tested the system and evaluated it with the following survey. One of the lecturer had 6 years of experience in teaching ultrasounds, the others all had more than 15 years of experience. All participants had experience in teaching how to use an ultrasound probe manually and all had used simulators before; however, none of them had ever used home-based training. Two of the participants considered practical training as the best way to teach ultrasounds; while the two other recommended a blended approach with both theoretical and practical learning. Table 1 summarises their responses to our questionnaire.

Overall, the participants thought that this tool could help apply theoretical anatomy learning to ultrasound practice (4±0.5). On the technical aspect, they all agreed that the system is easy to use; however, there is more diversity of the replies on the technical aspects such as the quality of the graphics (3.5±0.6) and the issues when using the system (3±0.8). The variety of the responses on the technical issues can be due to the fact that two of the participants encountered issues during the registration as they occluded the marker while grasping the simulated ultrasound probe. Specific to ultrasound education, the participants indicated the importance of scanning from multiple angles (3.75±1.0), but thought that the tactile feedback provided by the system was not realistic enough (2±1.2). On the educational value, the participants tended to say that the system was not good enough to be integrated in their student’s home-based training (2.5±1.3) as it is still an early prototype. The variety in these responses is likely due to the fact that some participants answered the question on the system as it currently is, while other based their answer on its potential with additional future developments.

The lecturers also gave free comments on the limitations and on what they would also like to add into the system. Globally they liked the idea of the system and feel it has great potential to be used as a training tool to learn cross sectional anatomy. They also said there is a gap in the market for this kind of system.

One of the features they would appreciate is the labelling and the display of instructions and comments related to the images. More precisely, they would like to have a temporary label related to the anatomical feature being displayed (“this is the brachial artery”) and on the movement the student is making (“moving the probe distally”) to learn the medical lingo. The labels would especially be useful as the student is learning without supervision and requires some sort of feedback.

To learn cross sectional anatomy, body tracking is not necessary—it would be sufficient to have only the virtual arm and the ultrasound images. In that case, the location of the probe to display the right image could be determine with a computer mouse instead of with body tracking. It could be included as a preliminary exercise to learn the anatomy before moving on to the ultrasound practice with the body tracking and the simulated transducer. Another possible feature would be to freeze the image to have time to study a specific image. An anatomy package could also be included to have a database of images to study.

Another comment is the importance of having a proper transducer replica to encourage correct holding of the transducer. The phone could be a good solution as it could be used to provide tactile or auditory feedback but could also be used as intermediate ultrasound display so that the student would not have to look at the screen instead of their arm.

An important feature to learn anatomy is the ability to scan in at least two imaging planes, more precisely cross section and trans-section. It is important to learn how to optimise the image by finding the best view. It would also be useful to have a more diverse and realistic database of ultrasound data for ultrasound practitioners. Furthermore, it would be important to be able to rest the arm while training because arm fatigue is one of the main source of injury for ultrasound practitioners. On the technical side, learners would like to have better performance in the display of the images, an easier registration at the beginning of the training session, and larger display of ultrasound images appear on the screen.

## 4. Discussion

One of the challenges in medical education is the necessity to give feedback to the student. Usually, the training is supervised by a specialist who is able to guide the student and offer feedback; however, requiring a specialist limits the generalisation of these training sessions. One of the comments from the lecturers also highlights the need of feedback during the training, especially because it aims as home-based training. The feedback could be instructions but also evaluations scores of the performances of the students. For instance, Pessin et al., highlighted the primary learning outcomes of ultrasound simulators as being scanning planes, spatial orientation, optimal image acquisition, and identification of anatomy and pathology. The high-fidelity simulators described in their review provide metric feedback allowing the student to compare their success against a standard [5].

The evaluation highlighted that it would be interesting to have the possibility to scan another person as it is more realistic to the sonographer experience; however, our system can only detect one person using the body tracking. Previous work has achieved body tracking of multiple individuals, and it would be a possibility to include that in this system to enhance the user experience. The body tracking would then assign an unique identification number to each person detected on the video [20], and the next step would be to assign one of the unique identification numbers to the patient and the other to the student training on how to perform a scan.

A notable limitation of the study is the small number of participants recruited to evaluate the system. As this was an initial feasibility assessment, we were not focused on achieving statistical power in our findings and instead focused on gathering early feedback from key stakeholders in this field to inform our future work. Furthermore, we only included experienced users in this validation steps because they have experience in using ultrasound and are able to identify how the system might succeed or fail to replicate real ultrasounds. Early-stage trainees lack this experience which allowed the experienced users to identify the limits of our system. Furthermore, the users all have experience in teaching ultrasounds and are able to identify what are the important learning points we need to focus on to improve the system; they have more knowledge on the ultrasound curriculum and on what the students need in their training.

Another limitation is the precision of the calibration between the two tracking methods which was not evaluated in this study. During the calibration procedure, the precision of tracking depends on the accuracy of the marker positioning by the participant on the different joints. Additionally, the ultrasound images were not recorded by a sonographer and are two-dimensional cine data. This can lead to inconsistencies between the position of the user and the display of the ultrasound images by the augmented reality system. As this was a preliminary study focused on feasibility assessment, the tracking error was not measured. That resulted in lower quality of the images which might have impacted the evaluation by the participants. However, the aim of the training system is to watch how the ultrasound image is modified when the user moves the probe along the arm (left to right/back to front); the accuracy between where the probe is located on the arm of the volunteer and the anatomy displayed was not the primary focus of this early feasibility study. In future work, we plan to improve on the accuracy of the system and quantify the error between the probe location during testing versus data recording.

Because of the system depends on pre-recorded ultrasound data, it is not possible to use it to learn image optimisation. However, future work could focus on creating a bigger image dataset with images taken at the same position but with different gain/focus/depth which would allow the student to practice this feature.

At last, the evaluation by the participants also highlights that the lack of force feedback and of the weight of a proper simulated probe is a limitation to the realism of the training experience. Towards this end, we made a 3D printed dummy probe (from the .fbx file of the virtual probe) which could be a solution for students who have access to a 3D printer; otherwise, sticking the marker on a mobile phone is also a good alternative as it has a rectangular shape similar to a probe and also adds some weight which provide a more realistic training. We understand that neither of these options are ideal, but they can easily be implemented by students to provide an initial solution.

## 5. Conclusions

This article described the development of a at-home ultrasound training system based on augmented reality. The objective was to develop a low-cost training system to teach cross sectional anatomy and the handling of an ultrasound probe that required only a computer with webcam and a printed marker. The concept was assessed by surgeons, anesthetist, and anatomy students, and the system was tested by ultrasound lecturers. Our evaluation highlighted the need for this type of system for ultrasound education, and the potential of our system to teach cross-sectional anatomy. Our system is however, preliminary, and requires further development to teach the complexities of handling of an ultrasound probe. From these initial findings, we reiterate the potential benefit of AR for anatomy training and ultrasound probe manipulation, provided that the tracking runs more smoothly and a simulated object is used for the probe.

## Figures and Tables

**Table 1:** Medical ultrasound lecturers feedback.

No.	Questions	Answers
Anatomy:		
6	In your opinion, could a tool like this help learning cross-sectional anatomy? [Yes/No]	Yes: 4 / No: 0
7	Could a tool like this help apply theoretical anatomy learning to ultrasound practice? 1–5 (1 strongly disagree, 3 don’t know, 5 to strongly agree)	mean: 4.25 (sd: 0.5)
8	Could a student use a tool like this to learn how to identify anatomical structures? [Yes/No]	Yes: 4 / No: 0
Technical: 1–5 (1 strongly disagree, 3 unsure, 5 to strongly agree)		
9	The graphics/visuals are realistic enough for home training?	mean: 3.5 (sd: 0.6)
10	The system is easy to use?	mean: 4 (sd: 0)
11	The system ran without issues/problems?	mean: 3 (sd: 0.8)
12	Being able to view the ultrasound images from multiple angles is useful?	mean: 3.75 (sd: 1.0)
13	The simulated probe provides tactile feedback realistic enough for a home-based training tool?	mean: 2 (sd: 1.2)
Educational Value: 1–5 (1 strongly disagree, 3 unsure, 5 to strongly agree)		
14	Do you feel this simulator is good enough for home training?	mean: 3 (sd: 0)
15	Would you include this as part of your students’ home-based training?	mean: 2.5 (sd: 1.3)
Free comments: Limitations and what they would like to add		

## Data Availability

The described software is freely available here: https://github.com/doughtmw/AR-Ultrasound-Training (accessed on 18 October 2022). Additional data is available on request from the corresponding author.
